# Dyport: dynamic importance-based biomedical hypothesis generation benchmarking technique

**DOI:** 10.1186/s12859-024-05812-8

**Published:** 2024-06-13

**Authors:** Ilya Tyagin, Ilya Safro

**Affiliations:** 1 Center for Bioinformatics and Computational Biology, University of Delaware, Newark, DE 19713 USA; 2 Department of Computer and Information Sciences, University of Delaware, Newark, DE 19716 USA

**Keywords:** Hypothesis Generation, Literature-based Discovery, Link Prediction, Benchmarking, Natural Language Processing

## Abstract

**Background:**

Automated hypothesis generation (HG) focuses on uncovering hidden connections within the extensive information that is publicly available. This domain has become increasingly popular, thanks to modern machine learning algorithms. However, the automated evaluation of HG systems is still an open problem, especially on a larger scale.

**Results:**

This paper presents a novel benchmarking framework Dyport for evaluating biomedical hypothesis generation systems. Utilizing curated datasets, our approach tests these systems under realistic conditions, enhancing the relevance of our evaluations. We integrate knowledge from the curated databases into a dynamic graph, accompanied by a method to quantify discovery importance. This not only assesses hypotheses accuracy but also their potential impact in biomedical research which significantly extends traditional link prediction benchmarks. Applicability of our benchmarking process is demonstrated on several link prediction systems applied on biomedical semantic knowledge graphs. Being flexible, our benchmarking system is designed for broad application in hypothesis generation quality verification, aiming to expand the scope of scientific discovery within the biomedical research community.

**Conclusions:**

Dyport is an open-source benchmarking framework designed for biomedical hypothesis generation systems evaluation, which takes into account knowledge dynamics, semantics and impact. All code and datasets are available at: https://github.com/IlyaTyagin/Dyport.

## Introduction

Automated hypothesis generation (HG, also known as Literature Based Discovery, LBD) has gone a long way since its establishment in 1986, when Swanson introduced the concept of “Undiscovered Public Knowledge” [1]. It pertains to the idea that within the public domain, there is a significant abundance of information, allowing for the uncovering of implicit connections among various pieces of information. There are many systems developed throughout the years, which incorporate different reasoning methods: from concept co-occurrence in scientific literature [2, 3] to the advanced deep learning-based algorithms and generative models (such as BioGPT [4] and CBAG [5]). Examples include but are not limited to probabilistic topic modeling over relevant papers [6], semantic inference [7], association rule discovery [8], latent semantic indexing [9], semantic knowledge network completion [10] or human-aware artificial intelligence [11] to mention just a few. The common thread running through these lines of research is that they are all meant to fill in the gaps between pieces of existing knowledge.

The evaluation of HG is still one of the major problems of these systems, especially when it comes to fully automated large-scale general purpose systems (such as IBM Watson Drug Discovery [12], AGATHA [10] or BioGPT [4]). For these, a massive assessment (that is normal in the machine learning and general AI domains) performed manually by the domain experts is usually not feasible and other methods are required.

One traditional evaluation approach is to make a system “rediscover” some of the landmark findings, similar to what was done in numerous works replicating well-known connections, such as: *Fish Oil *$\leftrightarrow$ *Raynaud’s Syndrome* [13], *Migraine *$\leftrightarrow$ *Magnesium* [13] or *Alzheimer *$\leftrightarrow$ *Estrogen* [14]. This technique is frequently used even in a majority of the recently published papers, despite of its obvious drawbacks, such as very limited number of validation samples and their general obsolesce (some of these connections are over 30 years old). Furthermore, in some of these works, the training set is not carefully chosen to include only the information published prior the discovery of interest which turns the HG goal into the information retrieval task.

Another commonly used technique is based on the time-slicing [10, 15], when a system is trained on a subset of data prior to a specified cut-off date and then evaluated on the data from the future. This method addresses the weaknesses of previous approach and can be automated, but it does not immediately answer the question of how significant or impactful the connections are. The lack of this information may lead to deceiving results: many connections, even recently published, are trivial (especially if they are found by the text mining methods) and do not advance the scientific field in a meaningful way.

A related area that faces similar evaluation challenges is Information Extraction (IE), a field crucial to enabling effective HG by identifying and categorizing relevant information in publicly available data sources. Within the realm of biomedical and life sciences IE, there are more targeted, small-scale evaluation protocols such as the BioCreative competitions [16], where the domain experts provide curated training and test datasets, which allows participants to refine and assess their systems within a controlled environment. While such targeted evaluations as conducted in BioCreative are both crucial and insightful, they inherently lack the scope and scale needed for the evaluation of expansive HG systems.

The aforementioned issues emphasize the critical need for research into effective, scalable evaluation methods in automated hypothesis generation. Our primary interest is in establishing an effective and sustainable benchmark for large-scale, general-purpose automated hypothesis generation systems within the biomedical domain. We seek to identify substantial, non-trivial insights, prioritizing them over mere data volume and ensuring scalability with respect to ever-expanding biocurated knowledge databases. We emphasize the significance of implementing sustainable evaluation strategies, relying on constantly updated datasets reflecting the latest research. Lastly, our efforts are targeted towards distinguishing between hypotheses with significant impact and those with lesser relevance, thus moving beyond trivial generation of hypotheses to ensuring their meaningful contribution to scientific discovery.

### Our contribution

- We propose a high quality benchmark dataset Dyport for hypothesis prediction systems evaluation. It incorporates information extracted from a number of biocurated databases. We normalize all concepts to the unified format for seamless integration and each connection is supplied with rich metadata, including timestamp information to enable time-slicing.
- We introduce an evaluation method for the impact of connections in time-slicing paradigm. It will allow to benchmark HG systems more thoroughly and extensively by assigning an importance weight to every connection over the time. This weight represents the overall impact a connection makes on future discoveries.
- We demonstrate the computational results of several prediction algorithms using the proposed benchmark and discuss their performance and quality.

We propose to use our benchmark to evaluate the quality of HG systems. The benchmark is designed to be updated on a yearly basis. Its structure facilitates relatively effortless expansion and reconfiguration by users and developers.

## Background and related work

Unfortunately, the evaluation in the hypothesis generation field is often coupled with the systems to evaluate and currently not universally standardized. If one would like to compare the performance of two or more systems, they need to understand their training protocol to instantiate models from scratch and then test them on the same data they used in their experiment.

This problem is well known and there are attempts to provide a universal way to evaluate such systems. For example, OpenBioLink [17] is designed as a software package for evaluation of link prediction models. It supports time-slicing and contains millions of edges with different quality settings. The authors describe it as “highly challenging” dataset that does not include “trivially predictable” connections, but they do not provide a quantification of difficulty nor range the edges accordingly.

Another attempt to set up a large-scale validation of HG systems was performed in our earlier work [18]. The proposed methodology is based on the semantic triples extracted from SemMedDB [19] database and setting up a cut date for training and testing. Triples are converted to pairs by removing the “verb” part from each *(subject-verb-object)* triple. For the test data, a list of “highly cited” pairs is identified, which is based on the citation counts from SemMedDB, MEDLINE and Semantic Scholar. Only connections occurring in papers published after the cut date and cited over 100 times are considered. It is worth mentioning that this approach is prone to noise (due to SemMedDB text mining methods) and also skewed towards the discoveries published closer to the cut-date, since the citations accumulate over time.

One more aspect of the proposed approach relates to the quantification and detection of scientific novelty. Research efforts range from protein design domain studies [20] to analyzing scientific publications through their titles [21] or using manual curation in combination with machine learning [22]. However, none of these techniques were integrated into a general purpose biomedical evaluation framework, where the novelty would be taken into account.

Currently, Knowledge Graph Embeddings (KGE) are becoming increasingly popular and the hypothesis generation problem can be formulated in terms of link prediction in knowledge graphs. Knowledge Graphs often evaluate the likelihood of a particular connection with the scoring function of choice. For example, TransE [23] evaluates each sample with the following equation:

$$\begin{aligned} s(h, r, t) = \Vert \textbf{h} + \textbf{r} - \textbf{t} \Vert , \end{aligned}$$

where *h* is the embedding vector of a head entity, *r* is the embedding vector of relation, *t* is the embedding vector of a tail entity and $||\cdot ||$ denotes the L1 or L2 norm.

These days KGE-based models are of interest to the broad scientific community, including researchers in the drug discovery field. Recently they carefully investigated the factors affecting the performance of KGE models [24] and reviewed biomedical databases related to drug discovery [25]. These publications, however, do not focus on any temporal information nor attempt to describe the extracted concept associations quantitatively. We also aim to fill in this currently existing gap in our current work.

## Methods

### Glossary

- $c_i$—concept in some arbitrary vocabulary;
- $m(\cdot )$—function that maps a concept $c_i$ to the subset of corresponding UMLS CUI. The result is denoted by $m_i =m(c_i)$. The $m_i$ is not necessarily a singleton. We will somewhat abuse the notation by denoting $m_i$ a single or any of the UMLS terms obtained by mapping $c_i$ to UMLS.
- $m(\cdot ,\cdot )$—function that maps pairs of $c_i$ and $c_j$ into the corresponding set of all possible UMLS pairs $m_i$ and $m_j$. Recall that the mapping of $c_i$ to UMLS may not be unique. In this case $|m(c_i,c_j)| = |m(c_i)|\cdot |m(c_j)|$.
- $(m_i, m_j)$—a pair of UMLS CUIs, which is extracted as a co-occurrence from MEDLINE records. It also represents an edge in network *G* and is cross-referenced with biocurated databases;
- *D*—set of pairs $(m_i, m_j)$ extracted from biocurated databases;
- *P*—set of pairs $(m_i, m_j)$ extracted from MEDLINE abstracts;
- *E*—set of cross-referenced pairs $(m_i, m_j)$, such that $E = D \cap P$;
- *G*—dynamic network, containing temporal snapshots $G_t$, where *t*—timestamp (year);
- $\hat{G}_t$—snapshot of network *G* for a timestamp *t* only containing nodes from $G_{t-1}$.

The main unit of analysis in HG is a connection between two biomedical concepts, which we also refer to as “pair”, “pairwise interaction” or “edge” (in network science context when we will be discussing semantic networks). These connections can be obtained from two main sources: biomedical databases and scientific texts. Extracting pairs from biomedical databases is done with respect to the nature and content of the database: some of them already contain pairwise interactions, whereas others focus on more complex structures such as pathways which may contain multiple pairwise interactions or motifs (e.g., KEGG [26]). Extracting pairs from textual data is done via information retrieval methods, such as relation extraction or co-occurrence mining. In this work, we use the abstract-based co-occurrence approach, which is explained later in the paper.

### Method in summary

**Fig. 1:**
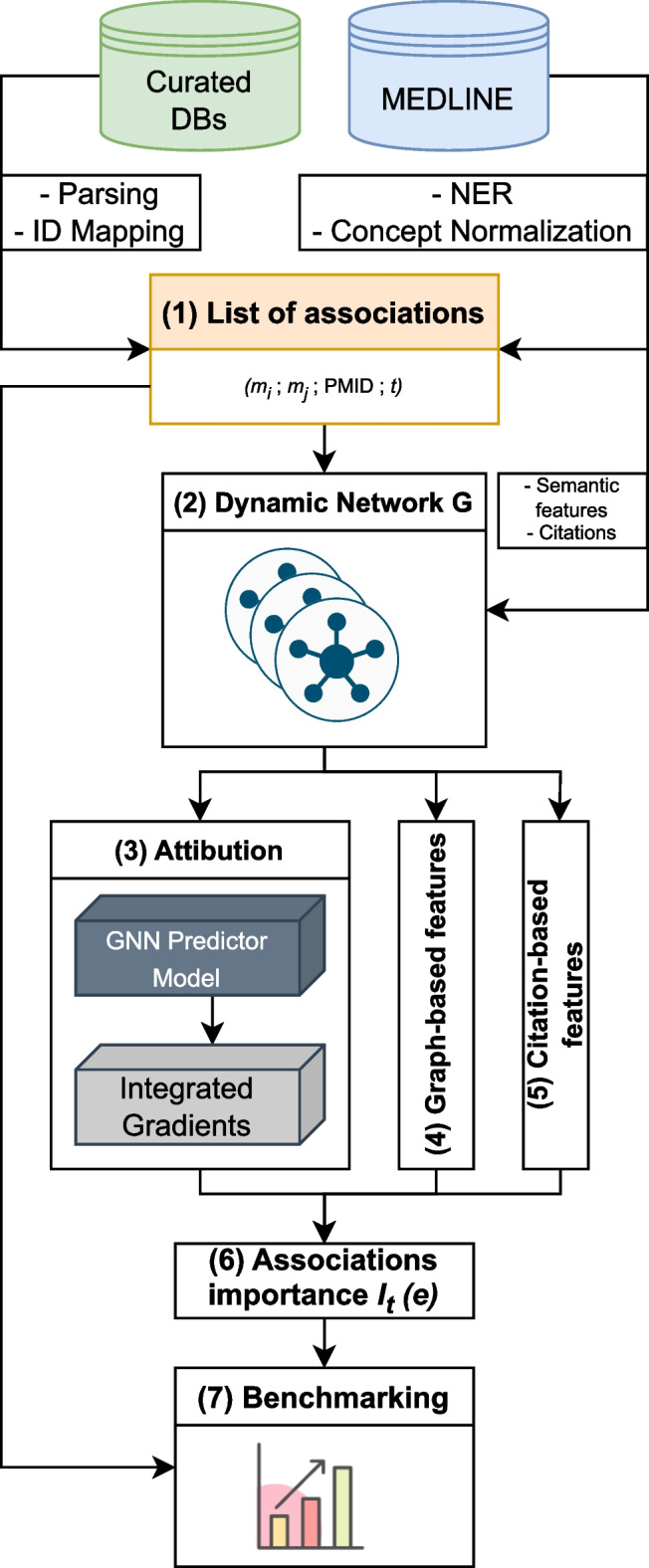
Summary of the HG benchmarking approach. We start with collecting data from Curated DBs and Medline, then process it: records from Curated DBs go through parsing, cleaning and ID mapping, MEDLINE records are fed into SemRep system, which performs NER and concept normalization. After that we obtain a list of UMLS CUI associations with attached PMIDs and timestamps (TS). This data is then used to construct a dynamic network *G*, which is used to calculate the importance measure *I* for edges in the network. At the end, edges $e \in G$ with their corresponding importance scores $I_t(e)$ are added to the benchmark dataset

The HG benchmarking pipeline is presented in Fig. 1. The end goal of the pipeline is to provide a way to evaluate any end-to-end hypothesis generation system trained to predict potential pairwise associations between biomedical instances or concepts.

We start with collecting pairwise entity associations from a list of biocurated databases, which we then normalize and represent as pairs of UMLS [27] terms $(m_i, m_j)$. The set of these associations is then cross-referenced with scientific abstracts extracted from MEDLINE database, such that for each pair $(m_i, m_j)$ we keep all PubMed identifiers (PMID) that correspond to the paper abstracts in which $m_i$ and $m_j$ co-occured. As a result, there is a list of tuples (step 1, Fig. 1) $(m_i, m_j, \text {PMID}, t)$, where *t* is a timestamp for a given PMID extracted from its metadata. We then split this list into a sequence $\{E_t\}$ according to the timestamp *t*. In this work *t* is taken with a yearly resolution.

Each individual $E_t$ can be treated as an edgelist, which yields an edge-induced network $G_t$ constructed from edges $(m_i, m_j) \in E_t$. It gives us a sequence of networks $G = \{G_t\}$ (step 2, Fig. 1), which is then used to compute the *importance* of individual associations in $E_t$ with different methods.

The main goal of importance is to describe each edge from $E_t$ using additional information. The majority of it comes from the future network snapshot $G_{t+1}$, which allows us to track the *impact* that a particular edge had on the network in the future. The predictive impact is calculated with an attribution technique called Integrated Gradients (IG) (step 3, Fig. 1). Structural impact is calculated with graph-based measures (such as centrality) (step 4, Fig. 1) and citation impact is calculated with respect to how frequently edges are referenced in the literature after their initial discovery (step 5, Fig. 1).

All the obtained scores are then merged together to obtain a ranking $I_t(e)$ (step 6, Fig. 1), where $e \in E_t$ for all edges from a snapshot $G_t$. Finally, this ranking is used to perform stratified evaluation of how well hypothesis generation systems perform in discovering connections with different importance values (step 7, Fig. 1).

### Databases processing and normalization

We begin by gathering the links and relationships from publicly available databases, curated by domain experts. *We ensure that all pairwise concept associations we utilize are from curated sources. For databases like STRING, which compile associations from various channels with differing levels of confidence, we exclusively select associations derived from curated sources.*

Ensuring correct correspondence of the same concepts from diverse databases is highly crucial. Therefore, we also conduct mapping of all concepts to UMLS CUI (Concept Unique Identifier). Concepts, which identifiers cannot be mapped to UMLS CUI, are dropped. In our process, we sometimes encounter situations where a concept $c_{i}$, may have multiple mappings to UMLS CUIs, i.e., $|m_i|=k>1$ for $m_i = m(c_i)$. To capture these diverse mappings, we use the Cartesian product rule. In this approach, we take the mapping sets for both concepts $c_{i}$ and $c_{j}$, denoted as $m(c_{i})$ and $m(c_{j})$, and generate a new set of pairs encapsulating all possible combinations of these mappings. Essentially, for each original pair $(c_{i}, c_{j})$, we produce a set of pairs $m(c_{i}, c_{j})$ such that the cardinality of this new set equals the product of the cardinalities of the individual mappings. Let us say that $c_i$ has *k* different UMLS mappings and $c_j$ has *s*, then $|m(c_{1},c_{2})| = |m(c_{1})| \cdot |m(c_{2})| = k\cdot s$.

In other words, we ensure that every possible mapping of the original pair is accounted for, enabling our system to consider all potential pairwise interactions across all UMLS mappings. To this end, we have collected all pairs of UMLS CUI that are present in different datasets, forming a set *D*.

### Processing MEDLINE records

To match pairwise interactions extracted from biocurated databases to literature, we use records from MEDLINE database with their PubMed identifiers. These records, primarily composed of the titles and abstracts of scientific papers, are each assigned a unique PubMed reference number (PMID). They are also supplemented with rich metadata, which includes information about authors, full-text links (when applicable), and date of publication timestamps indicating when the record became publicly available. We process records with an NLM-developed natural language processing tool SemRep [28] to perform named entity recognition, concept mapping and normalization. To this end, we obtain a list of UMLS CUI for each MEDLINE record.

### Connecting database records with literature

The next step is to form connections between biocurated records and their corresponding mentions in the literature. With UMLS CUIs identified in the previous step, we track the instances where these CUIs are mentioned together within the same scientific abstract. Our method considers the simultaneous appearance of a pair of concepts, denoted as $m_i$ and $m_j$, within a single abstract to represent a co-occurrence. This co-occurrence may indicate a potential relationship between the two concepts within the context of the abstract. All the co-occurring pairs $(m_i, m_j)$, extracted from MEDLINE abstracts, form the set *P*.

No specific “significance” score is assigned to these co-occurrences at this point beyond their presence in the same abstract. Subsequently, these pairs are cross-referenced with pairs in biocurated databases. More specifically, for each co-occurrence $(m_i, m_j) \in P$ we check its presence in set *D*. Pairs not present in both sets *D* and *P* are discarded. This forms the set *E*:

1 $$\begin{aligned} E = D \cap P. \end{aligned}$$

This step validates each co-occurring pair, effectively reducing noise and confirming that each pair holds biological significance. Conversely, *E* can be described as a set of biologically relevant associations, with each element enriched by contextual information extracted from scientific literature. The procedure is described in [29] as *distant supervised annotation*.

### Constructing time-sliced graphs

After we find the set of co-occurrences in abstracts extracted from MEDLINE and cross-referenced with pairs in biocurated databases (set *E*), we split it based on the timestamps extracted from the abstracts metadata. The timestamps *t* are assigned to each PMID and are used to determine when they became publicly available. We use these timestamps to track how often was a pair of UMLS CUIs $(m_i, m_j)$ appearing in the biomedical literature over time. As a result, we have a list of biologically relevant cross-referenced UMLS CUI co-occurrences, each connected to all PMIDs containing them.

This list is then split into edge lists $E_t$, such that each edge list contains pairs $(m_i, m_j)$ added in or before year *t*. These edge lists are then transformed to dynamic network *G* with *T* snapshots:

$$\begin{aligned} G = \{G_t=(N_t, E_t)\}_{t=1}^T, \end{aligned}$$

where $N_t$ and $E_t$ represent the set of unique UMLS CUIs (nodes) and their cross-referenced abstract co-occurrences (edges), respectively, and *t* is the annual timestamp (time resolution can be changed as needed), such that $G_{t}$ is constructed from all MEDLINE records published before *t* (e.g., $t=2011$). All networks $G_{t}$ are simple and undirected.

For each timestamp *t*, $G_{t}$ represents a *cumulative* network, including all the information from $G_{t-1}$ and new information added in year *t*.

### Tracking the edge importance of time-sliced graphs

We enrich the proposed benchmarking strategy with the information about associations importance at each time step *t*. In the context of scientific discovery, the importance may be considered from several different perspectives, e.g., as an the influence of an individual finding on future discoveries. In this section we take three different perspectives into account and then combine them together to obtain a final importance score, which we later use to evaluate different hypothesis generation systems with respect to their ability to predict the important associations.

#### Integrated gradients pipeline

In this step we obtain the information about how edges from graph $G_t$ influence the appearance of new edges in $G_{t+1}$. For that we train a machine learning model, which is able to perform link predictions and then we use an attribution method called Integrated Gradients (IG).

In general, IG is used to understand input features importance with respect to the output a given predictor model produces. In case of link prediction problem, a model outputs likelihood of two nodes $m_i$ and $m_j$ being connected for a given network $G_t$. The input features for a link prediction model will include the adjacency matrix of $G_t$, $A_t$, and the predictions themselves can be drawn from a list of edges appearing in the next timestamp $t + 1$. If IG is applied to this particular problem, it will provide attribution values for each element of $A_t$, which can be reformulated as the importance of edges existing at the timestamp *t* with respect to their contribution to predicting the edges added at the next timestamp $t+1$. This could be interpreted as the *influence* of current dynamic network structural elements on the information that will be added in future.

*Link prediction problem* In our setting, the link prediction problem is formulated as following:

$$\begin{aligned} & \text {given: } G_{t} = (N_t, E_t)\\ & \text {predict: } (m_i, m_j) \ \forall m_i, m_j \in \hat{G}_{t+1} (N_t, \hat{E}_{t+1}). \end{aligned}$$

We note that predictions of edges $\hat{E}_{t+1}$ are performed only for nodes $N_t$ from the graph $G_t$ at year *t*.

*Adding Node and Edge Features*: To enrich the dynamic network *G* with non-redundant information extracted from text, we add node features and edge weights. Node features are required for Graph Neural Network-based predictor training, which we use in the proposed pipeline.

*Node features*: Node features are added to each $G_t$ by applying word2vec algorithm [30] to the corresponding snapshot of MEDLINE dataset obtained for a timestamp *t*. In order to perform cleaning and normalization, we replace all tokens in the input texts by their corresponding UMLS CUIs obtained at the NER stage. It significantly reduces the vocabulary size, automatically removing stop-words and enabling vocabulary-guided phrase mining [31]. It is important to note that each node *m* has a different vector representation for each time stamp *t*, which we can refer to as *n*2*v*(*m*, *t*).

*Edge features (weights)*: For simplicity, edge weights are constructed by counting the number of MEDLINE records mentioning a pair of concepts $e \in E_{t}$. In other words, for each pair $e = (m_i, m_j)$ we assign a weight representing the total number of mentions for a pair *e* in year *t*.

#### GNN training

We use a graph neural network-based encoder-decoder architecture. Its encoder consists of two graph convolutional layers [32] and produces an embedding for each graph node. Decoder takes the obtained node embeddings and outputs the sum of element-wise multiplication of encoded node representations as a characteristic of each pair of nodes.

#### Attribution

To obtain a connection between newly introduced edges $\hat{E}_{t+1}$ and existing edges $E_t$, we use an attribution method Integrated Gradients (IG) [33]. It is based on two key assumptions:

- *Sensitivity:* any change in input that affects the output gets a non-zero attribution;
- *Implementation Invariance:* attribution is consistent with the model’s output, regardless of the model’s architecture.

The IG can be applied to a wide variety of ML models as it calculates the attribution scores with respect to input features and not the model weights/activations, which is important, because we focus on relationships between the data points and not the model internal structure.

The integrated gradient (IG) score along $i^{th}$ dimension for an input *x* and baseline $x'$ is defined as:

2 $$\begin{aligned} IG_{i}(x) {::}{=} (x_i - x'_i) \int _{\alpha =0}^{1} \frac{ \partial F(x' + \alpha (x-x') )}{\partial x_i } d\alpha , \end{aligned}$$

where $\frac{\partial F(x)}{\partial x_i}$ is the gradient of *F*(*x*) along $i^{th}$ dimension. In our case, input *x* is the adjacency matrix of $G_t$ filled with 1*s* as default values (we provide all edges $E_t \in G_t$) and baseline $x'$ is the matrix of zeroes. As a result, we obtain an adjacency matrix $A(G_t)$ filled with attribution values for each edge $E_t$.

#### Graph-based measures

*Betweenness Centrality* In order to estimate the structural importance of selected edges, we calculate their betweenness centrality [34]. This importance measure shows the amount of information passing through the edges, therefore indicating their influence over the information flow in the network. It is defined as

3 $$\begin{aligned} C_B(e) = \sum _{s \ne t \in V} \frac{\sigma _{st}(e)}{\sigma _{st}}, \end{aligned}$$

where $\sigma _{st}$—the number of shortest paths between nodes *s* and *t*; $\sigma _{st}(e)$—the number of shortest paths between nodes *s* and *t* passing through edge *e*.

To calculate the betweenness centrality with respect to the future connections, we restrict the set of vertices *V* to only those, that are involved in future connections we would like to use for explanation.

*Eigenvector Centrality* Another graph-based structural importance metric we use is the eigenvector centrality. The intuition behind it is that a node of the network is considered important if it is close to other important nodes. It can be found as a solution of the eigenvalue problem equation:

4 $$\begin{aligned} Ax = \lambda x, \end{aligned}$$

where *A* is the network weighted adjacency matrix. Finding the eigenvector corresponding to the largest eigenvalue gives us a list of centrality values $C_E(v)$ for each vertex $v \in V$.

However, we are interested in edge-based metric, which we obtain by taking an absolute difference between the adjacent vertex centralities:

5 $$\begin{aligned} C_E(e) = |C_E(u) - C_E(v) |, \end{aligned}$$

where $e=(u,v)$. The last step is to connect this importance measure to time snapshot, which we do by taking a time-base difference between edge-based eigenvector centralities

6 $$\begin{aligned} C_{E_{\Delta t}}(e) = C_{E_{t+1}}(e) - C_{E_t}(e), \end{aligned}$$

This metric gives us the eigenvector centrality change with respect to future state of the dynamic graph ($t+1$).

*Second Order Jaccard Similarity* One more indicator of how important a particular newly discovered network connection is related to its adjacent nodes neighborhood similarity. The intuition is that more similar their neighborhood is, more trivial the connection is, therefore, it is less important.

We consider a second-order Jaccard similarity index for a given pair of nodes $m_i$ and $m_j$:

7 $$\begin{aligned} J_2(m_i, m_j) = \frac{|N_2(m_i) \cap N_2(m_j)|}{|N_2(m_i) \cup N_2(m_j)|} \end{aligned}$$

Second-order neighborhood of a node *u* is defined by:

8 $$\begin{aligned} N_2(u) = \bigcup _{w \in N(u)} N(w), \end{aligned}$$

where *w* iterates over all neighbors of *u* and *N*(*w*) returns the neighbors of *w*.

The second order gives a much better “resolution” or granularity for different connections compared to first-order neighborhood. We also note that it is calculated for a graph $G_{t-1}$ for all edges $\hat{E}_{t}$ (before these edges were discovered).

#### Literature-based measures

*Cumulative citation counts* Another measure of a connection importance is related to bibliometrics. At each moment in time for each targeted edge we can obtain a list of papers mentioning this edge.

We also have access to a directed citation network, where nodes represent documents and edges represent citations: edges connect one paper to all the papers that it cites. Therefore, the number of citations of a specific paper would equal to in-degree of a corresponding node in a citation network.

To connect paper citations to concepts connections, we compute the sum of citation counts of all papers mentioning a specific connection. Usually, the citation counts follow heavy-tailed distributions (e.g., power law) and counting them at the logarithmic scale is a better practice. However, in our case the citation counts are taken “as-is” to emphasize the difference between the number of citations and the number of mentions. This measure shows the overall citation-based impact of a specific edge over time. The citation information comes from the citation graph, which is consistent with the proposed dynamic network in terms of time slicing methodology.

#### Combined importance measure for ranking connections

To connect all the components of the importance measure *I* for edge *e*, we use the mean percentile rank (PCTRank) of each individual component:

9 $$\begin{aligned} I_t(e) = \frac{1}{|C|}\sum _{C_i \in C }{\text {PCTRank}(C_{i_t}(e))}, \end{aligned}$$

where $C_i$ is the importance component (one of the described earlier, *C*—set of all importance components). The importance measure is calculated for each individual edge in graph for each moment in time *t* with respect to its future (or previous) state $t+1$ (or $t-1$). Using the mean percentile rank guarantees that the component will stay within a unit interval. The measure *I* is used to implement an importance-based stratification strategy for benchmarking, as it is discussed in Results section.

## Results

In this section we describe the experimental setup and propose a methodology based on different stratification methods. This methodology is unique for the proposed benchmark, because each record is supplied with additional information giving a user more flexible evaluation protocol.

### Data collection and processing

#### Dynamic graph construction

**Fig. 2:**
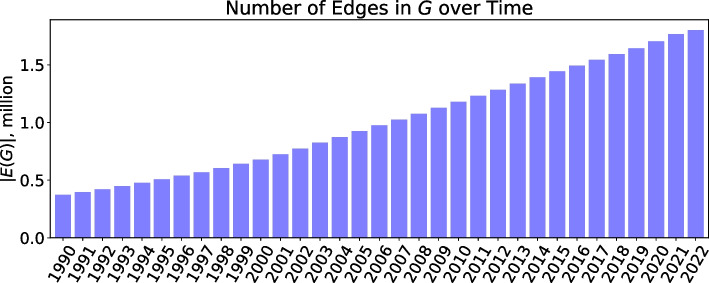
Number of edges in the network *G* over time. The numbers are reported in millions. Each edge represents a pair of cross-referenced UMLS CUI concepts $(m_i, m_j)$

The numbers of concepts and their associations successfully mapped to UMLS CUI $(m_i, m_j)$ from each dataset are summarized in Table 1. The number of associations with respect to time is shown in Fig. 2. It can be seen that the number of concept associations steadily and consistently grows for every subsequent year.

**Table 1:** Experimental databases included in the dataset

	Pairs	Concepts	Concept types
Database			
KEGG	730,214	22,306	Genes, Diseases, Chemicals
CTD	459,229	37,065	Genes, Diseases, Chemicals
DisGenNET	274,320	18,923	Genes, Diseases
DrugCentral	230,805	19,066	Genes, Diseases, Chemicals
RxNav	175,186	2804	Chemicals
STRING	63,904	9118	Proteins
Mentha	43,673	10,096	Proteins
GWAS	30,350	8905	Genes, Diseases

Reported numbers are for cross-referenced pairs of UMLS CUI concepts (edges) and the corresponding nodes from the network *G*

Data collection and aggregation is performed in the following pipeline:

1. All databases are downloaded in their corresponding formats such as comma-separated or Excel spreadsheets, SQL databases or Docker images.
2. All pairwise interactions in each database are identified.
3. From all these interactions we create a set of unique concepts, which we then map to UMLS CUIs. Concepts that do not have UMLS representations are dropped.
4. All original pairwise interactions are mapped with respect to the UMLS codes, as discussed in *Databases Processing and Normalization* section.
5. A set of all pairwise interactions is created by merging the mapped interactions from all databases.
6. This set is then used to find pairwise occurrences in MEDLINE.

Pairwise occurrences found in step 6 are used to construct the main dynamic network *G*. As it was mentioned earlier, *G* is undirected and non-attributed (we do not provide types of edges as they are much harder to collect reliably on large scale), which allows us to cover a broader range of pairwise interactions and LBD systems to test. Other pairwise interactions, which are successfully mapped to UMLS CUI, but are not found in the literature, can still be used. They do not have easily identifiable connections to scientific literature and do not contain temporal information, which make them a more difficult target to predict (will be discussed later).

#### Compound importance calculation

Once the dynamic graph *G* is constructed, we calculate the importance measure. For that we need to decide on three different timestamps:

1. Training timestamp: when the predictor models of interest are trained;
2. Testing timestamp: what moment in time to use to accumulate recently (with respect to step 1) discovered concept associations for models testing;
3. Importance timestamp: what moment in time to use to calculate the importance measure for concept associations from step 2.

To demonstrate our benchmark, we experiment with different predictive models. In our experimental setup, all models are trained on the data published prior to 2016, tested on associations discovered in 2016 and the importance measure *I* is calculated based on the most recent fully available timestamp (2022, at the time of writing) with respect to the PubMed annual baseline release. We note that, depending on the evaluation goals, other temporal splits can be used as well. For example, one can decide to evaluate the predictive performance of selected models on more recently discovered connections. For that, they may use the following temporal split: training timestamp—2020, testing timestamp—2021, importance timestamp—2022.

The importance measure *I* has multiple components, which are described in Methods section. To investigate their relationships and how they are connected to each other, we plot a Spearman correlation matrix showed in Table 2. Spearman correlation is used because only component’s *rank* matters in the proposed measure as all components are initially scaled differently.

**Table 2:** Correlation between components of the proposed importance measure

	IG	EC	BC	JC2	Ment.	Cit.
IG	1.000	0.162	0.045	0.102	0.022	0.154
EC	0.162	1.000	0.032	−0.028	0.091	0.119
BC	0.045	0.032	1.000	−0.010	−0.001	0.014
JC2	0.102	−0.028	−0.010	1.000	−0.050	0.018
Ment.	0.022	0.091	−0.001	−0.050	1.000	0.476
Cit.	0.154	0.119	0.014	0.018	0.476	1.000

Used abbreviations: IG—Integrated Gradients; EC—Eigenvector Centrality; BC—Betweenness Centrality; JC2—2nd order Jaccard Coefficient (negative); Ment.—Number of mentions; Cit.—Number of citations

### Evaluation protocol

In our experiments, we demonstrate a scenario for benchmarking hypothesis generation systems. All of the systems are treated as predictors capable of ranking true positive samples (which come from the dynamic network *G*) higher than the synthetically generated negatives. The hypothesis generation problem is formulated as binary classification with significant class imbalance.

#### Evaluation metric

The evaluation metric of choice for our benchmarking is Receiver Operating Characteristic (ROC) curve and its associated Area Under the Curve (AUC), which is calulated as:

10 $$\begin{aligned} AUC(f) = \frac{1}{|D^0| \cdot |D^1|} \sum _{t_0 \in D^0} \sum _{t_1 \in D^1} {\textbf {1}}[f(t_0) < f(t_1)] \end{aligned}$$

where ${\textbf {1}}$ is the indicator function that equals 1 if the score of a negative example $t_0$ is less than the score of a positive example $t_1$; $D^0$, $D^1$ are the sets of negative and positive examples, respectively. The ROC AUC score quantifies the model’s ability to rank a random positive higher than a random negative.

We note than the scores do not have to be within a specific range, the only requirement is that they can be compared with each other. In fact, using this metric allows us to compare purely classification-based models (such as Node2Vec logistic regression pipeline) and ranking models (like TransE or DistMult), even though the scores of these models may have arbitrary values.

#### Negative sampling

Our original evaluation protocol can be found in [10], which is called *subdomain recommendation*. It is inspired by how biomedical experts perform large-scale experiments to identify the biological instances of interest from a  large pool of candidates [35]. To summarize:

- We collect all positive samples after a pre-defined cut date. The data before this cut date is used for prediction system training.
- For each positive sample (subject-object pair) we generate *N* negative pairs, such that the subject is the same and the object in every newly generated pair has the same UMLS semantic type as the object in positive pair;
- We evaluate a selected performance measure (ROC AUC) with respect to pairs of semantic types (for example, gene-gene or drug-disease) to better understand domain specific differences.

For this experiment we set $N=10$ as a trade-off between the evaluation quality and runtime. It can be set higher if more thorough evaluation is needed.

### Baseline models description

To demonstrate how the proposed benchmark can be used to evaluate and compare different hypothesis generation system, we use a set of existing models. To make the comparison more fair, all of them are trained on the same snapshots of MEDLINE dataset.

#### AGATHA

The AGATHA is a general purpose HG system [10, 36] incorporates a multi-step pipeline, which processes the entire MEDLINE database of scientific abstracts, constructs a semantic graph from it and trains a predictor model based on transformer encoder architecture. Besides the algorithmic pipeline, the key difference between AGATHA and other link prediction systems is that AGATHA is an end-to-end hypothesis generation framework, where the link prediction is only one of its components.

#### Node2Vec

Node2Vec-based predictor is trained as suggested in the original publication [37]. We use a network purely constructed with text-mining-based methods.

#### Knowledge graph embeddings-based models

Knowledge Graph Embeddings (KGE) models are becoming increasingly popular these days, therefore we include them into our comparison. We use Ampligraph [38] library to train and query a list of KGE models: TransE, HolE, ComplEx and DistMult.

### Evaluation with different stratification

**Fig. 3:**
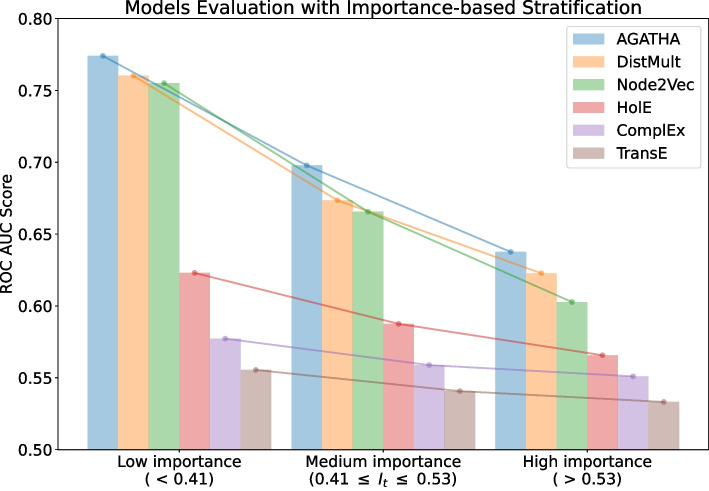
ROC AUC scores for different models trained on the same PubMed snapshot from 2015 and tested on semantic predicates added in 2016 binned with respect to their importance scores

**Fig. 4:**
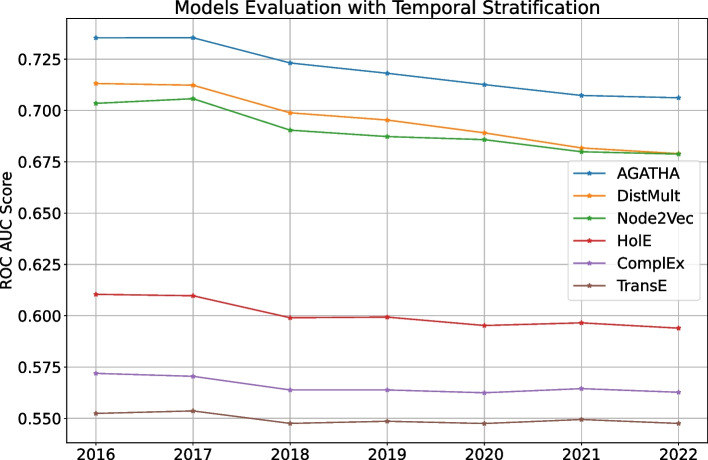
ROC AUC scores for different models trained on the same PubMed snapshot from 2015 and tested on semantic predicates added over time

The proposed benchmarking pipeline enables us to perform different kinds of systems evaluation and comparison with flexibility usually unavailable to other methods. Incorporating both temporal and importance information is helpful to identify trends in models behavior and extend the variety of criteria for domain experts when they decide on a best model suitable for their needs.

Below we present three distinct stratification methods and show how predictor models perform under different evaluation protocols. Even though we use the same performance metric (ROC AUC) across the board, the results differ substantially, suggesting that evaluation strategy plays a significant role in the experimental design.

#### Semantic stratification

Semantic stratification strategy is the natural way to benchmark hypothesis generation systems, when the goal is to evaluate performance in specific semantic categories. It is especially relevant to the subdomain recommendation problem, which defines our negative sampling procedure. For that we take the testing set of subject-object pairs and group them according to their semantic types and evaluate each group separately (Table 3).

**Table 3:** ROC AUC scores for different models trained on the same MEDLINE snapshot from 2015 and tested on semantic predicates added in the time frame between 2016 and 2022

Semantic pair	AGATHA	DistMult	Node2Vec	HolE	ComplEx	TransE	Dataset Size
Gene or Genome $\leftrightarrow$ Gene or Genome	0.604	0.581	0.577	0.547	0.539	0.527	606,573
Gene or Genome $\leftrightarrow$ Organic Chemical	0.732	0.693	0.660	0.598	0.560	0.552	359,436
Amino Acid, Peptide, or Protein $\leftrightarrow$ Gene or Genome	0.685	0.646	0.627	0.583	0.560	0.556	196,493
Organic Chemical $\leftrightarrow$ Organic Chemical	0.918	0.897	0.893	0.679	0.617	0.589	196,218
Gene or Genome $\leftrightarrow$ Pharmacologic Substance	0.701	0.672	0.643	0.583	0.550	0.539	109,604
Disease or Syndrome $\leftrightarrow$ Organic Chemical	0.876	0.871	0.855	0.673	0.607	0.566	100,111
Amino Acid, Peptide, or Protein $\leftrightarrow$ Organic Chemical	0.807	0.781	0.760	0.654	0.598	0.589	78,111
Organic Chemical $\leftrightarrow$ Pharmacologic Substance	0.890	0.870	0.871	0.656	0.590	0.565	54,340
Disease or Syndrome $\leftrightarrow$ Disease or Syndrome	0.833	0.826	0.822	0.636	0.574	0.555	38,467
Disease or Syndrome $\leftrightarrow$ Gene or Genome	0.680	0.678	0.612	0.570	0.547	0.526	32,549

#### Importance-based stratification

The next strategy is based on the proposed importance measure *I*. This measure ranks all the positive subject-object pairs from the test set and, therefore, can be used to split them into equally-sized bins, according to their importance score. In our experiment, we split the records into three bins, representing low, medium and high importance values. Negative samples are split accordingly. Then each group is evaluated separately. The results of this evaluation are presented in Fig. 3.

The results indicate that the importance score *I* could also reflect the *difficulty* of making a prediction. Specifically, pairs that receive higher importance scores tend to be more challenging for the systems to be identified correctly. In models that generally exhibit high performance (e.g., DistMult), the gap in ROC AUC scores between pairs with low importance scores and those with high importance scores is especially pronounced. The best model in this list is AGATHA as it utilizes the most nuanced hypothesis representation, namely, its transformer architecture is trained to leverage not only node embeddings but also to account for the non-overlapping neighborhoods of concepts.

#### Temporal stratification

The last strategy shows how different models trained once perform *over time*. For that we fix the training timestamp on 2015 and evaluate each models on testing timestamps from 2016 to 2022. For clarity, we do not use importance values for this experiment and only focus on how the models perform over time *on average*. The results are shown in Fig. 4.

Figure 4 highlights how predictive performance gradually decays over time for every model in the list. This behavior can be expected: the gap between training and testing data increases over time, which makes it more difficult for models to perform well as time goes by. Therefore, it is a good idea to keep the predictor models up-to-date, which we additionally discuss in the next section.

## Discussion

We divide the discussion into separate parts: topics related to evaluation challenges and topics related to different predictor model features. We also describe the challenges and scope for the future work at the end of the section.

### Evaluation-based topics

#### Data collection and processing challenges

The main challenge of this work comes from the diverse nature of biomedical data. This data may be described in many different ways and natural language may not be the most commonly used. Our results indicate that a very significant part of biocurated connections “flies under the radar” of text-mining systems and pipelines due to several reasons:

1. Imperfections of text-mining methods;
2. Multiple standards to describe biomedical concepts;
3. The diversity of scientific language: many biomedical associations (e.g. gene-gene interactions may be primarily described in terms of co-expression);
4. Abstracts are not enough for text mining [39].

The proposed methodology for the most part takes the lowest common denominator approach: we discard concepts not having UMLS representations and associations not appearing in PubMed abstracts. However, our approach still allows us to extract a significant number of concept associations and to use them for quantitative analysis. We should also admit that the aforementioned phenomenon of biomedical data discrepancy leads us to some interesting results, which we discuss below.

#### Different nature of biomedical DBs and literature-extracted data

The experiment clearly indicates significant differences between different kinds of associations with respect their corresponding data sources in models performance comparison. For this experiment we take one of the evaluated earlier systems (AGATHA 2015) and run the semantically-stratified version of benchmark collected from three different data sources:

1. Proposed benchmark dataset: concept associations extracted from biocurated databases with cross-referenced literature data;
2. Concept associations extracted from biocurated databases, but which we could not cross-reference with literature data;
3. Dataset composed of associations extracted with a text mining framework (SemRep).

Datasets (1) and (3) were constructed from associations found in MEDLINE snapshot from 2020. For dataset (2) it was impossible to identify the time connections were added, therefore the cut date approach was not used. All three datasets were downsampled with respect to the proposed benchmark (1), such that the number of associations is the same across all of them.

The results of this experiment are shown in Table 4. It is evident that associations extracted from biocurated databases (1) and (2) propose a more significant challenge for a text-mining-based system. Cross-referencing from literature makes sure that similar associations can be at least discovered by these systems at the training time, therefore, the AGATHA performance on dataset (1) is higher compared to dataset (2). These results may indicate that biocurated associations, which cannot be cross-referenced, belong to a different data distribution, and, therefore, purely text mining-based systems fall short due to the limitations of the underlying information extraction algorithms.

**Table 4:** AGATHA-2015 model performance (ROC AUC) evaluated on different data sources with the same cut-off date (where possible)

Semantic Pair	Text mining	Benchmark	Non-cross-ref DBs	Dataset size
Gene or Genome $\leftrightarrow$ Gene or Genome	0.858	0.612	0.530	42625
Gene or Genome $\leftrightarrow$ Organic Chemical	0.910	0.733	0.575	27060
Organic Chemical $\leftrightarrow$ Organic Chemical	0.905	0.922	0.679	15081
Amino Acid, Peptide, or Protein $\leftrightarrow$ Gene or Genome	0.897	0.695	0.591	14542
Gene or Genome $\leftrightarrow$ Pharmacologic Substance	0.901	0.702	0.592	7843
Disease or Syndrome $\leftrightarrow$ Organic Chemical	0.900	0.856	0.660	7612
Amino Acid, Peptide, or Protein $\leftrightarrow$ Organic Chemical	0.906	0.820	0.564	6072
Organic Chemical $\leftrightarrow$ Pharmacologic Substance	0.898	0.890	0.616	4070
Disease or Syndrome $\leftrightarrow$ Disease or Syndrome	0.853	0.854	0.666	2893
Disease or Syndrome $\leftrightarrow$ Gene or Genome	0.847	0.690	0.575	2442
Non-stratified ROC AUC	0.886	0.715	0.579	130240

Database records lacking literature cross-references (column 3) were randomly selected due to unavailability of temporal information for them

### Models-related topics

#### Text mining data characteristics

**Fig. 5:**
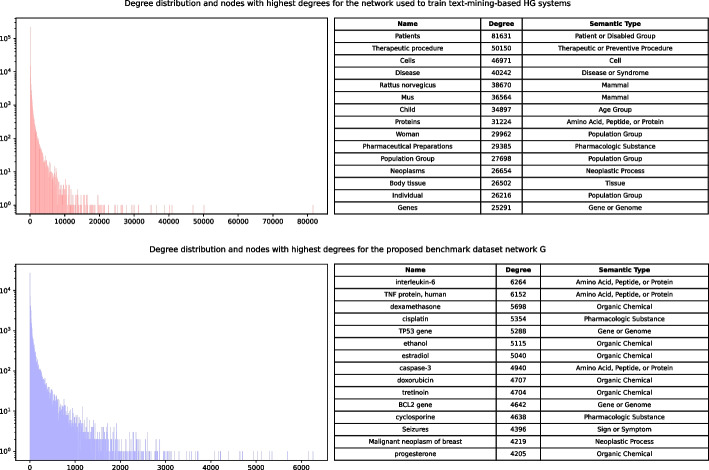
Degree distributions and nodes with highest degrees for two networks: the one used for training of text-mining-based predictor models (red, top) and the network *G* from the proposed benchmark dataset (blue, bottom)

In order to demonstrate the differences between biologically curated and text mining-based knowledge, we can consider their network representations.

The network-based models we show in this work are trained on text-mining-based networks, which are built on top of semantic predicates extracted from a NLP tool SemRep. This tool takes biomedical text as input and extracts triples *(subject-verb-object)* from the text and performs a number of additional tasks, such as:

- Named Entity Recognition
- Concept Normalization
- Co-reference Resolution

and some others. This tool operates on UMLS Metathesaurus, one of the largest and most diverse biomedical thesaurus, including many different vocabularies.

The main problem of text-mining tools like SemRep is that they tend to produce noisy (and often not quite meaningful from the biomedical prospective) data. As a result, the underlying data that is used to build and validate literature-based discovery systems may not represent the results that domain experts expect to see.

However, these systems are automated and, therefore, are widely used as a tool to extract information from literature in uninterrupted manner. Then this information is used for training different kinds of predictors (either rule-based, statistical or deep learning).

To demonstrate this phenomenon, we compare two networks, where nodes are biomedical terms and edges are associations between them. The difference between them lies in their original data source, which is either:

1. PubMed abstracts processed with SemRep tool;
2. Biocurated databases, which connections are mapped to pairs of UMLS CUI terms and cross-referenced with MEDLINE records.

Connections from the network (2) are used in the main proposed benchmarking framework (network *G*). The comparison is shown in Fig. 5 as a degree distribution of both networks. We can see that network (1) has a small number of very high-degree nodes. These nodes may affect negatively to the overall predictive power of any model using networks like (1) as a training set, because they introduce a large number of “shortcuts” to the network, which do not have any significant biological value. We also show the top most high-degree nodes for both networks. For the network (1), *all* of them appear to be very general and most of them (e.g. “Patients” or “Pharmaceutical Preparations”) can be described as noise. Network (2), in comparison, contain real biomedical entities, which carry domain-specific meaning.

#### Training data threshold influence

As the Temporal Stratification experiment in the Results section suggests, the gap between training and testing timestamps plays a noticeable role in models predictive performance.

To demonstrate this phenomena from a different perspective, we now fix the *testing timestamp* and vary the training timestamp. We use two identical AGATHA instances, but trained on different MEDLINE snapshots: 2015 and 2020. The testing timestamp for this experiment is 2021, such that none of the models has access to the test data.

The results shown in Table 5 illustrate that having more recent training data does not significantly increase model’s predictive power for the proposed benchmark. This result may be surprising, but there is a possible explanation: a model learns the patterns from the training data distribution and that data distribution stays consistent for both training cut dates (2015 and 2020). However, that does not mean that the data distribution in the benchmark behaves the same way. In fact, it changes with respect to both data sources: textual and DB-related.

**Table 5:** ROC AUC comparison between two AGATHA models trained on different MEDLINE snapshots: 2015 and 2020. A-15(20) stands for AGATHA 2015(20)

Semantic pair	A-15	A-20
Amino Acid, Peptide, or Protein $\leftrightarrow$ Gene or Genome	0.676	0.676
Amino Acid, Peptide, or Protein $\leftrightarrow$ Organic Chemical	0.772	0.774
Disease or Syndrome $\leftrightarrow$ Disease or Syndrome	0.838	0.849
Disease or Syndrome $\leftrightarrow$ Neoplastic Process	0.831	0.838
Disease or Syndrome $\leftrightarrow$ Organic Chemical	0.872	0.884
Gene or Genome $\leftrightarrow$ Gene or Genome	0.600	0.605
Gene or Genome $\leftrightarrow$ Organic Chemical	0.718	0.725
Gene or Genome $\leftrightarrow$ Pharmacologic Substance	0.691	0.692
Organic Chemical $\leftrightarrow$ Organic Chemical	0.907	0.913
Organic Chemical $\leftrightarrow$ Pharmacologic Substance	0.908	0.916
Non-stratified ROC AUC	0.691	0.695

#### Semantic types role in predictive performance

Another aspect affecting models predictive performance is having access to domain information. Since we formulate the problem as subdomain recommendation, knowing concept-domain relationships may be particularly valuable. We test this idea by injecting semantic types information into the edge type for tested earlier Knowledge Graph Embedding models. As opposed to classic link prediction methods (such as node2vec), Knowledge Graph modeling was designed around typed edges and allows this extension naturally.

Results in Table 6 show that semantic type information provides a very significant improvement for models predictive performance.

**Table 6:** ROC AUC scores comparison (non-stratified) between KGE-based models with and without semantic types information (ST) added to the training set

	ComplEx	DistMult	HolE	TransE
No ST	0.564	0.680	0.595	0.549
ST	0.693	0.697	0.718	0.678

#### Large language models for scientific discovery

**Fig. 6:**
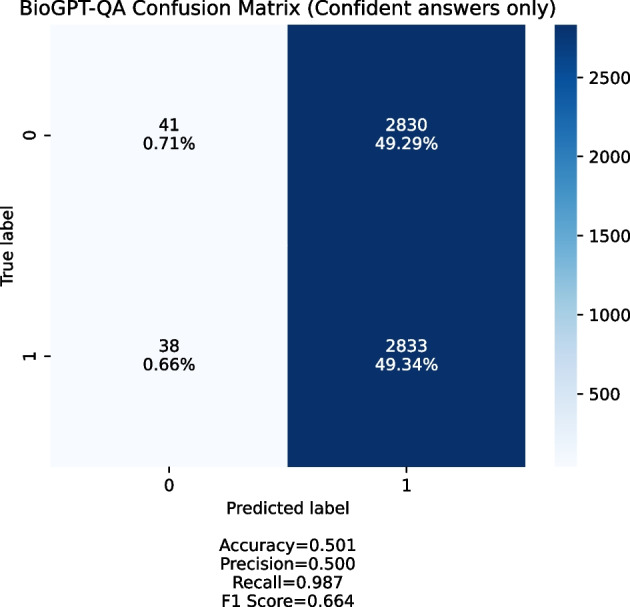
Confusion matrix obtained by the BioGPT-QA model. Only confident answers (Yes/No) were taken into account

Recent advances in language model development raised a logical question about usefulness of these models in scientific discovery, especially in biomedical area [40]. Problems like drug discovery, drug repurposing, clinical trial optimization and many others may benefit significantly from systems trained on a large amount of scientific biomedical data.

Therefore, we decide to test how one of these systems would perform in our benchmark. We take one of the recently released generative pre-trained transformer models BioGPT [4] and run a set of test queries.

BioGPT model was chosen due to the following reasons:

- It is recently released (2022);
- It includes fine-tuned models, which show good performance on downstream tasks;
- It is open source and easily accessible.

We use a BioGPT-QA model to perform the benchmarking, because it was fine-tuned on PubMedQA [41] dataset and outputs the answer as yes/maybe/no, which is easy to parse and represent as a (binary) classifier output.

The question prompt was formulated as the following: “Is there a relationship between <term 1> and <term 2>?”. PubMedQA format also requires a context from a PubMed abstract, which does not exist in our case, because it is a discovery problem. However, we supply an abstract-like context, which is constructed by concatenating term definitions extracted from UMLS Metathesaurus for both source and target terms.

A sample prompt looks like this: *“Is there a relationship between F1-ATPase and pyridoxal phosphate? context: F1-ATPase—The catalytic sector of proton-translocating ATPase complexes. It contains five subunits named alpha, beta, gamma, delta and eta. pyridoxal phosphate—This is the active form of VITAMIN B6 serving as a coenzyme for synthesis of amino acids, neurotransmitters (serotonin, norepinephrine), sphingolipids, aminolevulinic acid...”*

When we ran the experiment, we noticed two things:

- BioGPT is often not confident in its responses, which means that it outputs “maybe” or two answers (both “yes” and “no”) for about 40% of the provided queries;
- The overwhelming majority of provided queries are answered positively when the answer is confident.

Figure 6 shows a confusion matrix for queries with confident answer. We generate the queries set with 1:1 positive to negative ratio. Most of the answers BioGPT-QA provides are positive, which means that the system produces too many false positives and is not usable in the discovery setting.

### Challenges in benchmarking for hypothesis generation

**Binary interactions.** Not every discovery can be represented as a pair of terms, but this is something that most of biomedical graph-based knowledge discovery systems work with. It is a significant limitation of the current approach and a motif discovery is a valid potential direction for future work. Moreover, many databases represent their records as binary interactions [42–46], which can be easily integrated into a link prediction problem.

**Directionality.** Currently, our choice for pairwise interactions is to omit the directionality information to allow more systems to be evaluated with our framework and cover more pairwise interactions. Directionality is an important component of pairwise interactions, especially when they have types and are formulated in a predication form as a triple:*(subject-predicate-object)*. Currently, we omit the *predicate* part and only keep pairs of terms for easier generalization. In many cases, a uni-directional edge $i\rightarrow j$ does not imply non-existence of $i\leftarrow j$. Moreover, in the low-dimensional graph representation construction it is clearly preferable to use undirected edges in our context due to the scarcity of biomedical information. Another caveat is that the tools that detect the logical direction of the predicate in the texts are not perfect [47]. The information about each particular direction can still be recovered from the underlying cross-referencing citations.

**Concept normalization**. UMLS is a powerful system combining many biomedical vocabularies together. However, it has certain limitations, such as relatively small number of proteins and chemical compounds. We also observe that many UMLS terms are never covered in the scientific abstracts, even though they exist in the Metathesaurus. This limits the number of obtainable interactions significantly. However, UMLS covers many areas of biomedicine, such as genes, diseases, proteins, chemicals and many others and also provides rich metadata. In addition, NLM provides software for information extraction. There are other vocabularies, which have greater coverage in certain areas (e.g., UniProt ID for proteins or PubChem ID for chemicals), but their seamless integration into a heterogeneous network with literature poses additional challenges that will be gradually addressed in the future work.

## Conclusions

We have developed and implemented a comprehensive benchmarking system Dyport for evaluating biomedical hypothesis generation systems. This benchmarking system is advancing the field by providing a structured and systematic approach to assess the efficacy of various hypothesis generation methodologies.

In our pipeline we utilized several curated datasets, which provide a basis in testing the hypothesis generation systems under realistic conditions. The informative discoveries have been integrated into the dynamic graph on top of which we introduced the quantification of discovery importance. This approach allowed us to add a new dimension to the benchmarking process, enabling us to not only assess the accuracy of the hypotheses generated but also their relevance and potential impact in the field of biomedical research. This quantification of discovery importance is a critical step forward, as it aligns the benchmarking process more closely with the practical and applied goals of biomedical research.

We have demonstrated the use case of several graph-based link prediction systems’ verification and concluded that such testing is way more productive than traditional link prediction benchmarks. However, the utility of our benchmarking system extends beyond these examples. We advocate for its widespread adoption to validate the quality of hypothesis generation, aiming to broaden the range of scientific discoveries accessible to the wider research community. Our system is designed to be inclusive, welcoming the addition of more diverse cases.

Future work includes integration of the benchmarking process in the hypothesis system visualization [48], spreading to other than biomedical areas [49], integration of novel importance measures, and healthcare benchmarking cases.

## Appendix A: Incorporated technologies

To construct the benchmark, we propose a multi-step pipeline, which requires several key technologies to be used. For the text mining part, we use SemRep [28] and gensim [50] implementation of word2vec algorithm. For further stages involving graph learning, we utilize Pytorch Geometric framework and Captum explainability library.

*UMLS (Unified Medical Language System)* [27] is one of the fundamental technologies provided by NLM, which consolidates and disseminates essential terminology, taxonomies, and coding norms, along with related materials, such as definitions and semantic types. UMLS is used in the proposed work as a system of concept unique identifiers (CUI) bringing together terms from different vocabularies.

*SemRep *[47] is an NLM-developed software, performing extraction of semantic predicates from biomedical texts. It also has the named entity recognition (NER) capabilities (based on MetaMap [31] backend) and automatically performs entity normalization based on the context.

*Word2Vec* [30] is an approach for creating efficient word embeddings. It was proposed in 2013 and is proven to be an excellent technique for generating static (context-independent) latent word representations. The implementation used in this work is based on gensim [50] library.

*Pytorch geometric (PyG)* [51] library is built on top of Pytorch framework focusing on graph geometric learning. It implements a variety of algorithms from published research papers, supports arbitrary-scaled graphs and is well integrated into Pytorch ecosystem. We use PyG to train a graph neural network (GNN) for link prediction problem, which we explain in more detail in methods section.

*Captum *[52] package is an extension of Pytorch enabling the explainability of many ML models. It contains attribution methods, such as saliency maps, integrated gradients, Shapley value sampling and others. Captum is supported by PyG library and used in this work to calculate attributions of the proposed GNN.

## Appendix B: Incorporated data sources

We review and include a variety of biomedical databases, containing curated connections between different kinds of entities.

*KEGG (Kyoto Encyclopedia of Genes and Genomes)* [26] is a collection of resources for understanding principles of work of biological systems (such as cells, organisms or ecosystems) and offering a wide variety of entry points. One of the main components of KEGG is a set of pathway maps, representing molecular interactions as network diagrams.

*CTD (The Comparative Toxicogenomics Database)* [42] is a publicly available database focused on collecting the information about environmental exposures effects on human health.

*DisGenNET * [43] is a discovery platform covering genes and variants and their connections to human diseases. It integrates data from a list of publicly available databases and repositories and scientific literature.

*GWAS (Genome-Wide Association Studies)* [53] is a catalog of human genome-wide association studies, developed by EMBL-EBI and NHGRI. Its aim is to identify and systematize associations of genotypes with phenotypes across human genome.

*STRING *[54] is a database aiming to integrate known and predicted protein associations, both physical and functional. It utilizes a network-centric approach and assigns a confidence score for all interactions in the network based on the evidence coming from different sources: text mining, computational predictions and biocurated databases.

*DrugCentral* [44] is an online drug information resource aggregating information about active ingredients, indications, pharmacologic action and other related data with respect to FDA, EMA and PMDA-approved drugs.

*Mentha* [45] is an evidence-based protein interaction browser (and corresponding database), which takes advantage of International Molecular Exchange (IMEx) consortium. The interactions are curated by experts in compliance with IMEx policies enabling regular weekly updates. Compared to STRING, Mentha is focused on precision over comprehensiveness and excludes any computationally predicted records.

*RxNav* [46] is a web-service providing an integrated view on drug information. It contains the information from NLM drug terminology RxNorm, drug classes RxClass and drug-drug interactions collected from ONCHigh and DrugBank sources.

*Semantic scholar* [55] is a search engine and research tool for scientific papers developed by the Allen Institute for Artificial Intelligence (AI2). It provides rich metadata about publications which enables us to use Semantic Scholar data for network-based citation analysis.

## Declarations

### Availability of data and materials

The dataset(s), materials and code supporting the conclusions of this article is(are) available in the GitHub repository: https://github.com/IlyaTyagin/Dyport.

## References

[1] Swanson DR (1986). Undiscovered public knowledge. Libr Q.

[2] Swanson DR, Smalheiser NR, Torvik VI (2006). Ranking indirect connections in literature-based discovery: the role of medical subject headings. J Am Soc Inform Sci Technol.

[3] Peng Y, Bonifield G, Smalheiser N (2017). Gaps within the biomedical literature: Initial characterization and assessment of strategies for discovery. Front Res Metrics Anal.

[4] Luo R, Sun L, Xia Y, Qin T, Zhang S, Poon H, Liu T-Y (2022). Biogpt: generative pre-trained transformer for biomedical text generation and mining. Brief Bioinform.

[5] Sybrandt J, Safro I (2021). Cbag: conditional biomedical abstract generation. PLoS ONE.

[6] Sybrandt J, Shtutman M, Safro I. Moliere: automatic biomedical hypothesis generation system. In: Proceedings of the 23rd ACM SIGKDD. KDD ’17, 2017. pp. 1633–1642. ACM, New York, NY, USA. 10.1145/3097983.3098057. 10.1145/3097983.3098057 PMC5804740 29430330

[7] Sedler AR, Mitchell CS (2019). Semnet: using local features to navigate the biomedical concept graph. Front Bioeng Biotechnol.

[8] Hristovski D, Peterlin B, Mitchell JA, Humphrey SM (2005). Using literature-based discovery to identify disease candidate genes. Int J Med Inform.

[9] Gordon MD, Dumais S (1998). Using latent semantic indexing for literature based discovery. J Am Soc Inf Sci.

[10] Sybrandt J, Tyagin I, Shtutman M, Safro I. AGATHA: automatic graph mining and transformer based hypothesis generation approach. In: Proceedings of the 29th ACM international conference on information and knowledge management, 2020;2757–64.

[11] Sourati J, Evans J (2023). Accelerating science with human-aware artificial intelligence. Nat Hum Behav.

[12] Chen Y, Argentinis JE, Weber G (2016). IBM Watson: how cognitive computing can be applied to big data challenges in life sciences research. Clin Ther.

[13] Xun G, Jha K, Gopalakrishnan V, Li Y, Zhang A. Generating medical hypotheses based on evolutionary medical concepts. In: 2017 IEEE International conference on data mining (ICDM), pp. 535–44 (2017). 10.1109/ICDM.2017.63.

[14] Cameron D, Kavuluru R, Rindflesch TC, Sheth AP, Thirunarayan K, Bodenreider O. Context-driven automatic subgraph creation for literature-based discovery. J Biomed Inform. 2015;54:141–57. 10.1016/j.jbi.2015.01.014. 10.1016/j.jbi.2015.01.014 PMC4888806 25661592

[15] Sebastian Y, Siew E-G, Orimaye SO (2017). Learning the heterogeneous bibliographic information network for literature-based discovery. Knowl-Based Syst.

[16] Miranda A, Mehryary F, Luoma J, Pyysalo S, Valencia A, Krallinger M. Overview of drugprot biocreative vii track: quality evaluation and large scale text mining of drug-gene/protein relations. In: Proceedings of the seventh biocreative challenge evaluation workshop, 2021;11–21.

[17] Breit A, Ott S, Agibetov A, Samwald M (2020). OpenBioLink: a benchmarking framework for large-scale biomedical link prediction. Bioinformatics.

[18] Sybrandt J, Shtutman M, Safro I. Large-scale validation of hypothesis generation systems via candidate ranking. In: 2018 IEEE international conference on big data, 2018; 1494–1503. 10.1109/bigdata.2018.8622637. 10.1109/bigdata.2018.8622637 PMC9248026 35789222

[19] Kilicoglu H, Shin D, Fiszman M, Rosemblat G, Rindflesch TC (2012). Semmeddb: a pubmed-scale repository of biomedical semantic predications. Bioinformatics.

[20] Fannjiang C, Listgarten J (2024). Is novelty predictable?. Cold Spring Harb Perspect Biol.

[21] Jeon D, Lee J, Ahn J, Lee C (2023). Measuring the novelty of scientific publications: a fastText and local outlier factor approach. J Inform.

[22] Small H, Tseng H, Patek M (2017). Discovering discoveries: Identifying biomedical discoveries using citation contexts. J Inform.

[23] Bordes A, Usunier N, Garcia-Duran A, Weston J, Yakhnenko O. Translating embeddings for modeling multi-relational data. In: Advances in neural information processing systems, 2013; 2787–2795.

[24] Bonner S, Barrett IP, Ye C, Swiers R, Engkvist O, Hoyt CT, Hamilton WL (2022). Understanding the performance of knowledge graph embeddings in drug discovery. Artif Intell Life Sci..

[25] Bonner S, Barrett IP, Ye C, Swiers R, Engkvist O, Bender A, Hoyt CT, Hamilton WL (2022). A review of biomedical datasets relating to drug discovery: a knowledge graph perspective. Brief Bioinform.

[26] Kanehisa M, Sato Y, Kawashima M, Furumichi M, Tanabe M (2015). KEGG as a reference resource for gene and protein annotation. Nucleic Acids Res.

[27] Bodenreider O. The unified medical language system (umls): integrating biomedical terminology. Nucleic Acids Res. 2004;32(suppl_1):267–70. 10.1093/nar/gkh061 PMC308795 14681409

[28] Rindflesch TC, Fiszman M (2003). The interaction of domain knowledge and linguistic structure in natural language processing: interpreting hypernymic propositions in biomedical text. J Biomed Inform..

[29] Xing R, Luo J, Song T (2020). Biorel: towards large-scale biomedical relation extraction. BMC Bioinform.

[30] Mikolov T, Sutskever I, Chen K, Corrado GS, Dean J. Distributed representations of words and phrases and their compositionality. Adv Neural Inf Process Syst 2013;26.

[31] Aronson AR. Effective mapping of biomedical text to the umls metathesaurus: the metamap program. In: Proceedings of the AMIA symposium, 2001;p. 17. PMC2243666 11825149

[32] Welling M, Kipf TN. Semi-supervised classification with graph convolutional networks. In: Journal of international conference on learning representations (ICLR 2017), 2016.

[33] Sundararajan M, Taly A, Yan Q. Axiomatic attribution for deep networks. In: International conference on machine learning, pp. 3319–3328, 2017.

[34] Brandes U (2001). A faster algorithm for betweenness centrality. J Math Sociol.

[35] Aksenova M, Sybrandt J, Cui B, Sikirzhytski V, Ji H, Odhiambo D, Lucius MD, Turner JR, Broude E, Peña E, et al. Inhibition of the dead box rna helicase 3 prevents hiv-1 tat and cocaine-induced neurotoxicity by targeting microglia activation. J Neuroimmune Pharmacol. 2019;1–15. 10.1007/s11481-019-09885-8 PMC8048136 31802418

[36] Tyagin I, Kulshrestha A, Sybrandt J, Matta K, Shtutman M, Safro I. Accelerating covid-19 research with graph mining and transformer-based learning. In: Proceedings of the AAAI conference on artificial intelligence, 2022;36:12673–9.

[37] Grover A, Leskovec J. Node2vec: Scalable feature learning for networks. In: Proceedings of the 22nd ACM SIGKDD international conference on knowledge discovery and data mining. KDD ’16, 2016, pp. 855–864. Association for Computing Machinery, New York. 10.1145/2939672.2939754 . 10.1145/2939672.2939754 PMC5108654 27853626

[38] Costabello L, Bernardi A, Janik A, Pai S, Van CL, McGrath R, McCarthy N, Tabacof P. AmpliGraph: a library for representation learning on knowledge graphs, 2019. 10.5281/zenodo.2595043.

[39] Sybrandt J, Carrabba A, Herzog A, Safro I. Are abstracts enough for hypothesis generation? In: 2018 IEEE international conference on big data, 2018;1504–1513. 10.1109/bigdata.2018.8621974.

[40] Liu Z, Roberts RA, Lal-Nag M, Chen X, Huang R, Tong W (2021). Ai-based language models powering drug discovery and development. Drug Discovery Today.

[41] Jin Q, Dhingra B, Liu Z, Cohen WW, Lu X. Pubmedqa: a dataset for biomedical research question answering, 2019; arXiv preprint arXiv:1909.06146.

[42] Davis AP, Wiegers TC, Johnson RJ, Sciaky D, Wiegers J, Mattingly CJ (2022). Comparative toxicogenomics database (ctd): update 2023. Nucleic Acids Res.

[43] Piñero J, Ramírez-Anguita JM, Saüch-Pitarch J, Ronzano F, Centeno E, Sanz F (2019). Furlong LI The DisGeNET knowledge platform for disease genomics: 2019 update. Nucleic Acids Res.

[44] Ursu O, Holmes J, Knockel J, Bologa CG, Yang JJ, Mathias SL, Nelson SJ, Oprea TI (2016). DrugCentral: online drug compendium. Nucleic Acids Research.

[45] Calderone A, Castagnoli L, Cesareni G (2013). Mentha: a resource for browsing integrated protein-interaction networks. Nat Methods.

[46] Zeng K, Bodenreider O, Kilbourne J, Nelson SJ. Rxnav: a web service for standard drug information. In: AMIA annual symposium proceedings, 2006; vol. 2006, p. 1156. PMC1839335 17238775

[47] Kilicoglu H, Rosemblat G, Fiszman M, Shin D (2020). Broad-coverage biomedical relation extraction with SemRep. BMC Bioinform..

[48] Tyagin I, Safro I. Interpretable visualization of scientific hypotheses in literature-based discovery. BioCretive Workshop VII; 2021. https://www.biorxiv.org/content/10.1101/2021.10.29.466471v1.

[49] Marasco D, Tyagin I, Sybrandt J, Spencer JH, Safro I. Literature-based discovery for landscape planning, 2023. arXiv preprint arXiv:2306.02588.

[50] Rehurek R, Sojka P. Gensim-python framework for vector space modelling. NLP Centre, Faculty of Informatics, Masaryk University, Brno, Czech Republic 2011;3(2).

[51] Fey M, Lenssen JE. Fast graph representation learning with PyTorch Geometric. In: ICLR workshop on representation learning on graphs and manifolds, 2019.

[52] Kokhlikyan, N., Miglani, V., Martin, M., Wang, E., Alsallakh, B., Reynolds, J., Melnikov, A., Kliushkina, N., Araya, C., Yan, S., Reblitz-Richardson, O. Captum: a unified and generic model interpretability library for PyTorch, 2020.

[53] Sollis E, Mosaku A, Abid A, Buniello A, Cerezo M, Gil L, Groza T, Güneş O, Hall P, Hayhurst J, Ibrahim A, Ji Y, John S, Lewis E, MacArthur JL, McMahon A, Osumi-Sutherland D, Panoutsopoulou K, Pendlington Z, Ramachandran S, Stefancsik R, Stewart J, Whetzel P, Wilson R, Hindorff L, Cunningham F, Lambert S, Inouye M, Parkinson H, Harris L (2022). The NHGRI-EBI GWAS catalog: knowledgebase and deposition resource. Nucleic Acids Res..

[54] Szklarczyk D, Gable AL, Nastou KC, Lyon D, Kirsch R, Pyysalo S, Doncheva NT, Legeay M, Fang T, Bork P, Jensen LJ, von Mering C (2020). The STRING database in 2021: customizable protein-protein networks, and functional characterization of user-uploaded gene/measurement sets. Nucleic Acids Research.

[55] Fricke S (2018). Semantic scholar. J Med Lib Assoc: JMLA.

